# Indoor vs. Outdoor Walking: Does It Make Any Difference in Joint Angle Depending on Road Surface?

**DOI:** 10.3389/fspor.2020.00119

**Published:** 2020-09-18

**Authors:** Haruki Toda, Tsubasa Maruyama, Mitsunori Tada

**Affiliations:** ^1^Artificial Intelligence Research Center, National Institute of Advanced Industrial Science and Technology, Tokyo, Japan; ^2^Human Augmentation Research Center, National Institute of Advanced Industrial Science and Technology, Chiba, Japan

**Keywords:** ankle plantar flexion excursion, walking, outdoor environment, inertial measurement unit (IMU), motion capture (Mocap)

## Abstract

Measurement of the joint angle during walking in real-world environments facilitates comprehension of the adaptation strategy corresponding to road surfaces. This study investigated the differences between the joint angles in the lower limb when walking on flat road surfaces in indoor and outdoor environments. Ten healthy young males who walked on a carpet-lined corridor in the indoor environment and on an interlocking block pavement surface in the outdoor environment participated in the study. The joint angles of their lower limbs were measured using seven inertial measurement units, and the average and coefficient of variation (%CV) of the joint angular excursion in the two environments were evaluated. The %CVs of the ankle plantar flexion excursion in the early stance was 45% higher in the outdoor environment compared with that in the indoor, although the spatiotemporal parameters and joint angular excursion of the proximal joints showed no difference between the environments. Though the road surfaces were flat from a macroscopic point of view, the interlocking block pavement had stiffer and more irregular characteristics. The variability in the ankle plantar flexion motion in the early stance may be most likely affected by these surface characteristics in the real-world outdoor environment.

## Introduction

In daily life, people walk on various types of road surfaces, since walking is indispensable for promoting social life and health (Jacobs et al., 2008). However, in Japan, several falls occur owing to environmental factors such as the surface characteristics of outdoor environments (Niino et al., 2003). Thus, analyzing the physical behavior of walking on various road surfaces is important for understanding the walking strategy necessary to adapt to the different surface characteristics of outdoor environments.

Studies on gait analysis have primarily been performed in laboratories—where the pathways are clean, flat, and short—using optical motion capture systems (MoCap) (Winter, 1984; Kadaba et al., 1990). To simulate the effects of various terrains, the joint kinematics during walking have been evaluated on walkways with randomly placed wooden blocks beneath artificial grass (Thies et al., 2005a,b; Menant et al., 2009), compliant foam (MacLellan and Patla, 2016), and loose rock surface (Gates et al., 2012) constructed for research purpose in laboratories. When walking on such surfaces, the variability in the step width and stride time (Thies et al., 2005a,b), the peak joint angle, and the standard deviation (SD) of the hip, knee, and ankle across the gait cycle (Gates et al., 2012) increased, while the walking speed and stride length (Thies et al., 2005b; Menant et al., 2009) decreased. These studies revealed that the kinematic profiles during walking are adapted to the corresponding irregular road surface. However, the pathways in the laboratory were not as long as those in the outdoor environments, and the surface characteristics differ from those of the real-road surfaces because they were constructed for research purposes. Therefore, it was unclear whether the kinematic change during walking according to the irregular road surfaces obtained in these studies reproduce those in real outdoor environments.

Recent studies have attempted to quantitatively evaluate walking in an outdoor environment using inertial measurement units (IMUs). Specifically, the cadence and speed of daily walking (Weiss et al., 2013; Fasel et al., 2017), and the variability and stability of the acceleration waveform in outdoor conditions (Iosa et al., 2012a,b; Tamburini et al., 2018) have been evaluated from time-series acceleration data measured by IMUs. These studies have reported that the spatiotemporal parameters were affected by the walking environment (Iosa et al., 2012a,b; Fasel et al., 2017; Tamburini et al., 2018). These previous studies focused on the evaluation of walking in the outdoor environment by analyzing of the acceleration data, but the joint angles in the lower extremity were not evaluated when walking in the outdoor environment.

To overcome the limitation of optical MoCap systems, MoCap systems using IMUs attached to each body segment have been developed (Roetenberg et al., 2009; Seel et al., 2014). Maruyama et al. (2018) developed a real-time MoCap system using IMUs that can measure the position of the subjects as well as the joint angles. In previous studies, the accuracy of the IMU-based MoCap systems was evaluated, and it was confirmed to be excellent for hip, knee, and ankle joint angles in the sagittal plane during walking on a flat surface (Al-Amri et al., 2018; Maruyama et al., 2018). Therefore, by using an IMU-based MoCap system, the joint motion in the sagittal plane during walking in environments other than the laboratory can be investigated; this was not possible using conventional optical MoCap systems.

Measurement and analysis of the joint motion during walking on different types of road surfaces in the outdoor environment can facilitate comprehension of realistic adaptation strategy corresponding to various types of road surfaces. Hence, this study investigated the difference between the joint angles when walking on indoor and outdoor road surfaces by an IMU-based MoCap system. In daily life, people often walk on paved flat surfaces, and rarely walk on irregular road surfaces as constructed for research purposes in the previous studies (Gates et al., 2012; Blair et al., 2018; Dixon et al., 2018). Therefore, we hypothesized that the joint motions in the lower extremity were not affected by the flat road surface in real outdoor environments.

## Method

### Participants

Ten healthy young males (age: 24.1 ± 1.9 years, height: 1.70 ± 0.05 m, weight: 61.4 ± 8.3 kg) participated in this study. None of the subjects had any history of neuromuscular diseases, trauma, or orthopedic diseases. The experimental protocol was approved by the local ethical committee, and all the participants provided written informed consent before participating.

### Data Collection

**Figure 1:**
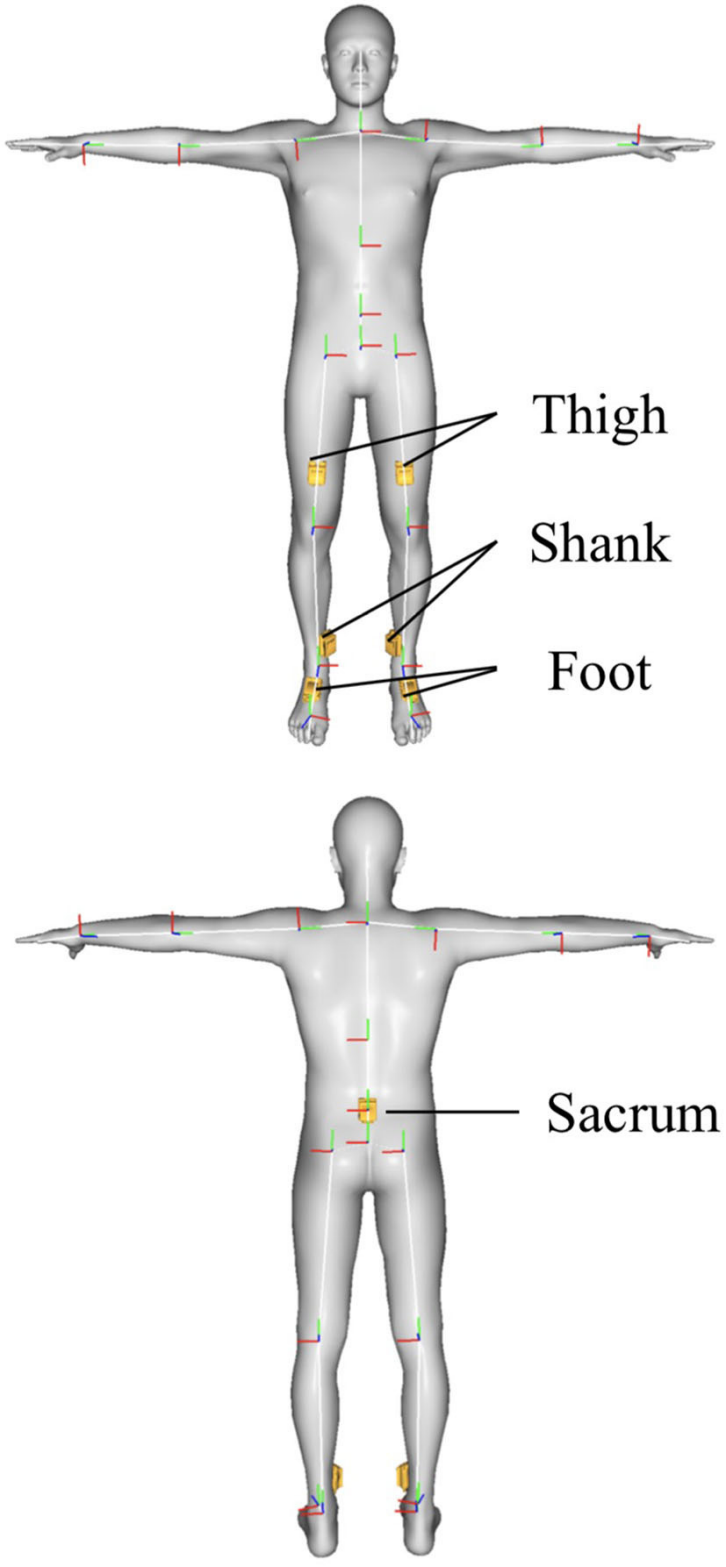
Placement of inertial measurement units on the whole-body model.

Each participant had seven IMUs (MTw; Xsens Technologies Inc., Enschede, Netherlands) attached to the sacrum, bilateral thigh, shank, and foot (Figure 1). Before the walking session, the participants were asked to adopt a reference pose for calibrating the IMU-based MoCap system, in which the IMU orientation relative to the corresponding body segment was determined. The subjects walked along a straight carpet-lined corridor in the indoor environment and on an interlocking block pavement surface in the outdoor environment. This is because the IMU-based MoCap system used in this study had been validated only in a laboratory with flat floor surfaces. The slope in the progression direction of the outdoor walkway was <1° as measured by a three-dimensional laser scanner (FOCUS^s^ 70; FARO Inc., Lake Mary, USA) (Yang et al., 2013). Although the road surfaces are flat from a macroscopic point of view, the interlocking block pavement is stiffer than the carpet, and have small irregularities due to the misalignment of the blocks (Hata et al., 2003).

**Figure 2:**
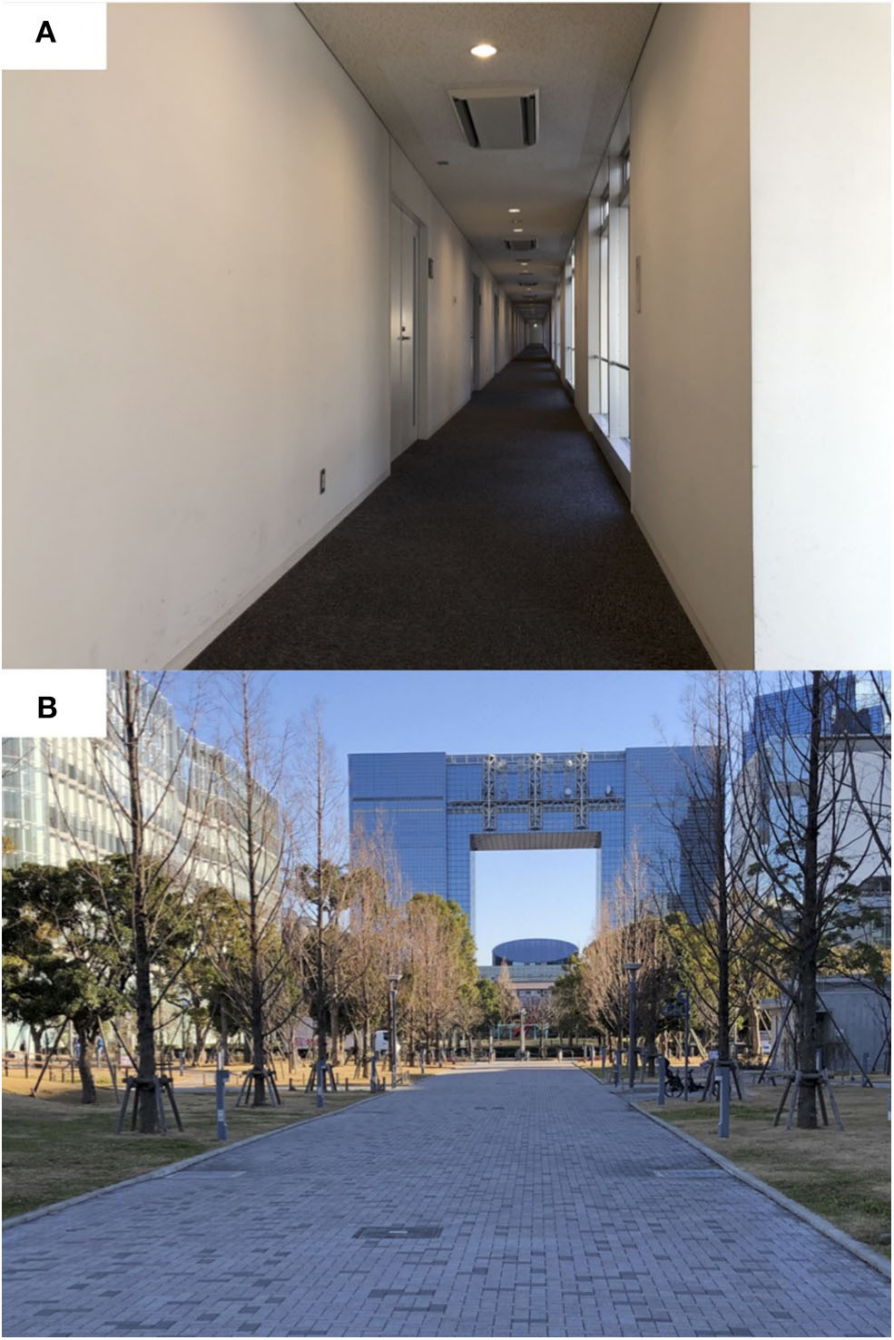
Photographs depicting **(A)** the corridor in the indoor environment and **(B)** the walkway in the outdoor environment where the measurements were performed. Subjects walked on a level surface and over an interlocking block pavement surface. The slope angles in the progression and lateral direction of the outdoor walkway were <1°.

The walking distance was ~90 m which was equal to the maximum length of the corridor in the indoor environment (Figure 2). All walking sessions were conducted at a self-selected preferred walking speed and with the same shoes (BioTF 02; Moonstar Inc., Fukuoka, Japan). The order of the two walking sessions was randomized. During these sessions, the data from the IMUs were sampled at 60 Hz, and the longest measurement duration was <5 min. Within this duration, the drift error of the IMU is negligible, as reported previously (Robert-Lachaine et al., 2017; Paulich et al., 2018). The errors of the angles relative to those measured using the optical MoCap system ranged from 2.0° ± 0.3° (ankle) to 10.9° ± 4.0° (hip) in the sagittal plane (Maruyama et al., 2018). The waveform similarities were also evaluated using the cross-correlation coefficient and were confirmed to range from 0.86 (ankle) to 0.97 (knee) under this measurement condition.

### Data Analysis

The joint angles of the hip, knee, and ankle in the sagittal plane, and the position of the center of mass (CoM) of the whole-body model were calculated using a posture-reconstruction plugin (Maruyama et al., 2018) running on DhaibaWorks—our self-developed motion analysis software (Endo et al., 2014). This plugin reconstructed the lower limb motion by combining the orientation data of each IMU and the individual body model with a link structure. The dimensions of the body model were estimated statistically from the participant's height and weight, based on the database of Japanese body dimensions (Endo et al., 2014).

**Figure 3:**
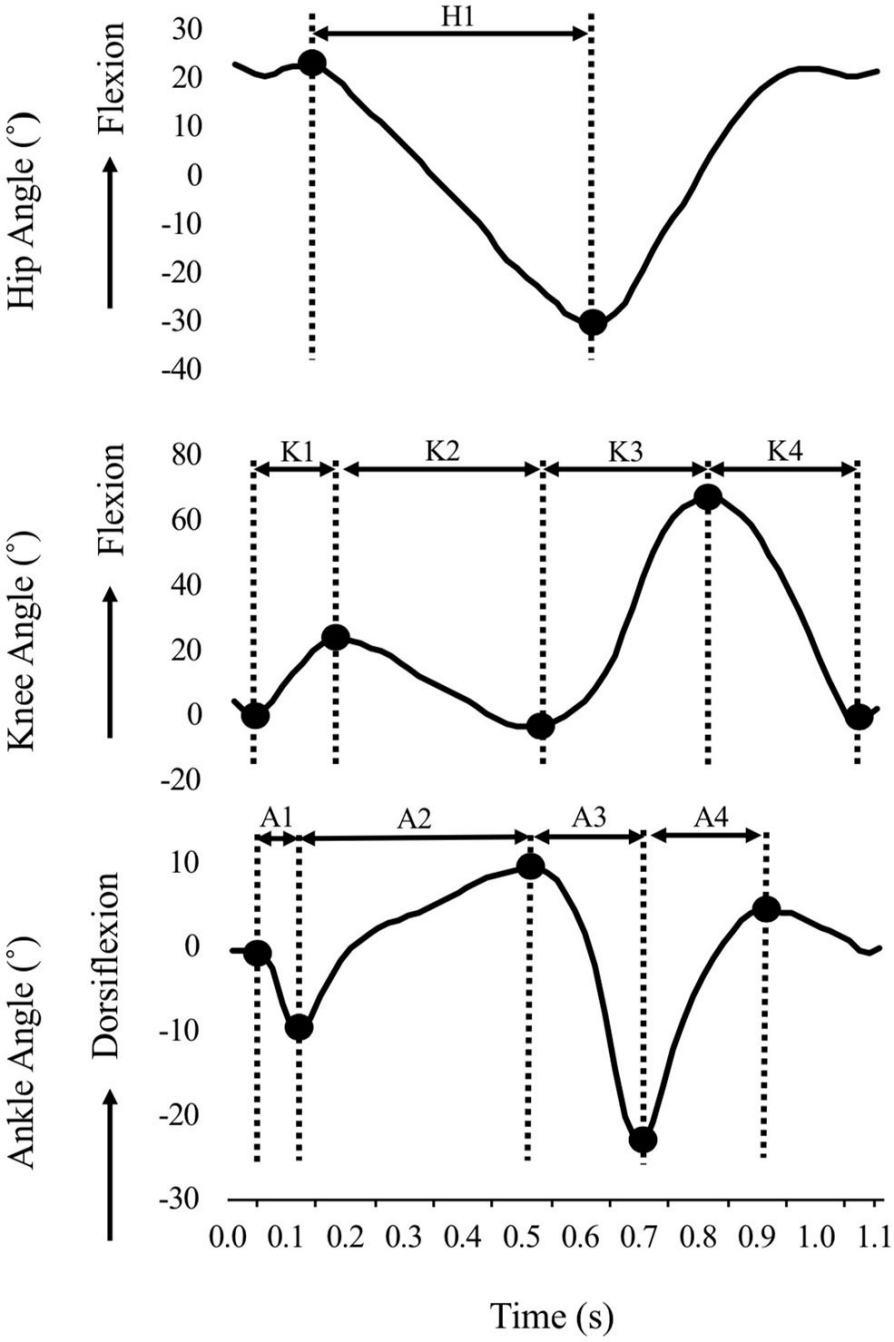
Graphical representation of joint angles of the hip, knee, and ankle in time series. Black dots indicate the key points in a gait cycle. The joint angular excursions were calculated from the amplitude of the displacements between these values. H1: Hip joint excursion; K1: Knee flexion excursion in early stance; K2: Knee extension excursion in mid-stance; K3: Knee flexion excursion in late stance; K4: Knee extension excursion in swing; A1: Ankle plantar flexion excursion in early stance; A2: Ankle dorsiflexion excursion in mid-stance; A3: Ankle plantar flexion excursion in late stance; A2: Ankle dorsiflexion excursion in swing.

Data for 30 strides during steady-state walking were extracted, as similarly performed in a previous study to analyze stride-to-stride kinematic variability (Dingwell and Cavanagh, 2001). In addition, the joint angular excursions were calculated from the amplitude of the displacements between the key points in a gait cycle (Figure 3). The mean and SD values were calculated across the gait cycle, and the coefficient of variation (%CV) was calculated as an index of the variability of the joint angular excursion, as follows:

$$\%CV=\frac{SD}{Mean}\;\times \;100$$

The walking speed (m/s) and cadence (step/min) were calculated using the Euclidean distance of the position of the CoM in the horizontal plane and using one gait cycle time, respectively. These values were calculated for 30 gait cycles and subsequently averaged.

The spatiotemporal parameters, joint angular excursion, and %CV were calculated using MATLAB R2018a (MathWorks Inc., Natick, USA).

### Statistical Analysis

Differences in mean and %CV of the spatiotemporal parameters and joint angular excursions of the hip, knee, and ankle of the subjects, between indoor and outdoor road surfaces, were analyzed using Wilcoxon signed ranks tests. Values of *p* < 0.05 were considered statistically significant. All data were analyzed using SPSS Statistics version 25.0 (SPSS Inc., Chicago, USA). The *r* values were calculated as effect sizes that are the magnitudes of the differences between the environments. The amplitudes of these values were classified as small (0.1 ≤ *r* < 0.3), moderate (0.3 ≤ *r* < 0.5), and large (0.5 ≤ *r*).

## Results

Table 1 shows the analysis results. The mean values of the spatiotemporal parameters and the joint angular excursions of the hip, knee, and ankle did not differ significantly between the environments.

**Table 1:** Mean and coefficient of variation (%CV) of the spatiotemporal parameters and joint angular excursions.

**Variable**	**Joint**	**Motion**	**Phase**	**Indoor**	**Outdoor**	***p-*value**	**Effect size**
Mean value							
Walking speed (m/s)				1.48 (1.35–1.58)	1.52 (1.35–1.60)	0.51	0.21
Cadence (step/min)				117.1 (111.6–118.4)	117.9 (111.2–119.7)	0.20	0.40
Angular excursion (°)	Hip	Excursion		48.1 (47.3–48.5)	50.8 (46.5–51.8)	0.58	0.18
	Knee	Flexion	Early stance	19.9 (18.2–22.9)	22.4 (20.3–24.1)	0.33	0.31
		Extension	Mid-stance	20.0 (17.7–22.2)	23.3 (21.8–24.7)	0.11	0.50
		Flexion	Late stance	64.8 (60.9–68.9)	66.2 (59.7–71.7)	0.33	0.31
		Extension	Swing	66.9 (63.2–67.1)	67.3 (62.8–71.3)	0.96	0.02
	Ankle	Plantar flexion	Early stance	9.3 (8.5–10.2)	8.0 (6.7–10.4)	0.45	0.24
		Dorsiflexion	Mid-stance	17.3 (15.9–17.4)	17.5 (14.2–17.9)	0.45	0.24
		Plantar flexion	Late stance	37.5 (34.5–38.2)	37.8 (34.0–39.4)	0.88	0.05
		Dorsiflexion	Swing	30.4 (26.8–35.4)	28.3 (25.3–28.6)	0.14	0.47
%CV (%)							
Walking Speed				3.1 (2.2–4.1)	3.0 (2.4–3.9)	0.95	0.02
Cadence				1.9 (1.5–2.1)	1.7 (1.4–1.9)	0.57	0.16
Angular excursion	Hip	Excursion		2.6 (2.4–2.9)	2.5 (2.3–2.6)	0.96	0.02
	Knee	Flexion	Early stance	8.5 (7.0–9.6)	10.1 (8.4–10.6)	0.96	0.02
		Extension	Mid-stance	8.6 (7.1–9.1)	8.5 (7.0–8.7)	0.45	0.24
		Flexion	Late stance	2.5 (2.2–3.0)	3.3 (2.9–3.6)	0.06	0.60
		Extension	Swing	2.4 (1.5–2.8)	2.8 (2.2–3.0)	0.24	0.37
	Ankle	Plantar flexion	Early stance	13.6 (11.3–16.9)	19.8 (17.4–21.2)	0.02^*^	0.76
		Dorsiflexion	Mid-stance	9.9 (8.0–10.8)	10.7 (9.6–12.3)	0.20	0.40
		Plantar flexion	Late stance	4.5 (3.1– 6.1)	5.0 (4.0–7.3)	0.29	0.34
		Dorsiflexion	Swing	6.4 (5.1–7.9)	9.2 (6.8–12.8)	0.14	0.47

*Values: central value (Lower quartile-Upper quartile)*.

* *Significant difference between the indoor and outdoor environments (p < 0.05)*.

The %CV of the plantar flexion excursion of the ankle in the early stance in the outdoor environment was 45% higher than that in the indoor environment. This difference yielded large effect size (*r* = 0.76). However, no statistically significant differences were observed in %CVs of the spatiotemporal parameters and the hip and knee joint angular excursions between the environments.

## Discussion

This study examined the differences in the joint angles of the lower extremity when walking on flat road surfaces in indoor and outdoor environments. Nevertheless, the %CV of the ankle joint angular excursion in early stance was confirmed to be higher in the outdoor environment, without any changes to the spatiotemporal parameters and the joint angular excursions of the hip and knee joints. These results did not support our hypothesis.

The difference observed in the %CV value between the indoor and outdoor environments indicates that the variability in the ankle plantar flexion excursion increases when walking in the outdoor environment. On the contrary, the walking environment did not influence the amplitude of the angular excursions of the hip, knee, and ankle joints. Previous studies performed in the laboratory reported that the joint angles of the hip, knee, and ankle increased when walking on a destabilizing loose rock surface (Gates et al., 2012) and an uneven surface (Blair et al., 2018; Dixon et al., 2018). In addition, the vertical CoM movement decreased with a large flexion motion of the trunk and lower extremity (Gates et al., 2012). These kinematic changes reflect motor control strategies to overcome perturbations imposed by the uneven road surface. The results of our study did not completely conform to those of these previous studies because the interlocking block pavement was flat compared with the previous studies (Gates et al., 2012; Blair et al., 2018; Dixon et al., 2018), although it had small irregularities due to the misalignment of the blocks. Nevertheless, we found that compared with the indoor environment, the interlocking block road in the outdoor environment leads to the increase in the variability in the ankle plantar flexion excursion in the early stance without affecting the variability of the joint angular excursion of the hip and knee.

The ankle plantar flexion motion in the early stance provides the contact of the foot with the ground. Therefore, adapting the plantar surface of the foot to the walking surface through this motion is important for stable walking on uneven terrain (Gates et al., 2013). In this study, subjects walked on the carpet-lined corridor in the indoor environment and the inter-rocking block pavement in the outdoor environment. Although both road surfaces were flat from a macroscopic point of view, the interlocking block pavement was stiffer than the carpet and had small irregularities due to the misalignment of the blocks (Hata et al., 2003). Thus, the variability of the ankle plantar flexion excursion, which provides the initial contact between the foot and the ground in early stance becomes large to adapt to the road surface in outdoor environments. We speculated that the proximal joints and the other phases were not affected by the surface characteristic using this adaptation in the healthy young subjects. On the other hand, the people with the ankle-foot orthosis and prosthetic foot (Gates et al., 2013) and the older people with peripheral muscle weakness (Menz et al., 2004) cannot be variably varied the ankle plantar flexion motion in the early stance. Even walking on the relatively flat surface in the outdoor environment, their proximal joints were likely to increase in the variability during the entire stance phase.

In addition to the surface characteristics, visual information also differed between the indoor corridor and outdoor open-field environments. The visual perturbation affected the step length (Iosa et al., 2012a,b) and width (Franz et al., 2015) and their variabilities (Thompson and Franz, 2017). In particular, these effects were larger among older adults than young people. In this study, the only variability of the ankle plantar flexion in the loading response that had a small association with the step width and length (Van Hedel et al., 2006) were affected by the walking environments. In young people, the effect of the difference in the visual information between the environments was small. We speculated that the difference in the variability in the ankle plantar flexion was due to the surface characteristics.

Despite the new revelations, our study has certain limitations. First, the walking motion was analyzed over only a single surface type in an outdoor environment, though in real life, people walk on various types of surfaces, from flat asphalt to irregular gravel. In this study, it is unclear which factors of the surface characteristics—stiffness or irregularity—affected the increase in the variability of the ankle plantar flexion excursion during loading response. Further research is necessary to evaluate the walking strategy corresponding to the characteristics of terrains in real outdoor environments. Second, all participants in this study were healthy young males. Previous studies clarified that the joint kinematics of the elderly were affected by the road surface more significantly than those of the young (Blair et al., 2018; Dixon et al., 2018). Further studies are needed to investigate the variability in the joint angular excursion during real outdoor walking for the elderly. Third, the small sample size and many comparison tests may produce a type I and II error, respectively. Nevertheless, the difference in the %CV of the ankle plantar flexion excursion between the environments had a large effect size that is a quantitative measure of the magnitude of the difference. Therefore, we speculated that the walking environments affected the %CV of the ankle plantar flexion excursion in the early stance.

To our knowledge, this is the first study that has investigated the differences in the joint motion during walking between the indoor and the real-world outdoor environments. The variability in the ankle plantar flexion in the early stance phase increased when walking in the outdoor environment, although the spatiotemporal parameters and joint angular excursion of the hip and knee joints were not different between the two walking environments. The measurement and analysis of the joint motion during walking in the real-world environment make it possible to reveal a more realistic adaptation strategy corresponding to the outdoor road surface. This study suggests that the variability of the ankle plantar flexion excursion during loading response becomes large to adapt to the road surface in the outdoor environment, without affecting the joint angular excursion of the hip and knee.

## Data Availability Statement

Datasets are available upon request to the corresponding author.

## Ethics Statement

The studies involving human participants were reviewed and approved by National Institute of Advanced Industrial Science and Technology. The patients/participants provided their written informed consent to participate in this study.

## Author Contributions

HT, TM, and MT conceived and designed the experiments and interpreted data. HT and TM performed the experiment. HT conducted data analysis and drafted the manuscript. TM and MT edited and revised the manuscript and approved the final version. All authors contributed to the article and approved the submitted version.

## Conflict of Interest

The authors declare that the research was conducted in the absence of any commercial or financial relationships that could be construed as a potential conflict of interest.
